# Biocompatible and Biodegradable Surfactants from Orange Peel for Oil Spill Remediation

**DOI:** 10.3390/molecules28155794

**Published:** 2023-08-01

**Authors:** Peng Soon Wang, Aqeel Ahmad, Masooma Nazar, Anisa Ur Rahmah, Muhammad Moniruzzaman

**Affiliations:** 1 Department of Chemical Engineering, Universiti Teknologi PETRONAS, Seri Iskandar 32610, Perak, Malaysia; peng_18002311@utp.edu.my (P.S.W.); aqeel.ahmad@utp.edu.my (A.A.); masooma_18000375@utp.edu.my (M.N.); 2 Department of Chemical Engineering, Universitas Muhammadiyah Surakarta, Kartasura 57162, Sukoharjo, Indonesia; aur744@ums.ac.id; 3 Center of Research in Ionic Liquids (CORIL), Universiti Teknologi PETRONAS, Seri Iskandar 32610, Perak, Malaysia

**Keywords:** orange peel, surfactant, dispersant effectiveness, toxicity, biodegradable, oil spill

## Abstract

Oil spill remediation plays a vital role in mitigating the environmental impacts caused by oil spills. The chemical method is one of the widely recognized approaches in chemical surfactants. However, the most commonly used chemical surfactants are toxic and non-biodegradable. Herein, two biocompatible and biodegradable surfactants were synthesized from orange peel using the ionic liquid 1-butyl-3-methylimidazolium chloride (BMIMCl) and organic solvent dimethylacetamide (CH3CN(CH3)2) as reaction media. The acronyms SOPIL and SOPOS refer to the surfactants prepared with BMIMCl and dimethylacetamide, respectively. The surface tension, dispersant effectiveness, optical microscopy, and emulsion stability test were conducted to examine the comparative performance of the synthesized surfactants. The Baffled flask test (BFT) was carried out to determine the dispersion effectiveness. The toxicity test was performed against zebrafish (Danio rerio), whereas the closed bottle test (CBT) evaluated biodegradability. The results revealed that the critical micelle concentration (CMC) value of SOPIL was lower (8.57 mg/L) than that of SOPOS (9.42 mg/L). The dispersion effectiveness values for SOPIL and SOPOS were 69.78% and 40.30%, respectively. The acute toxicity test demonstrated that SOPIL was ‘practically non-toxic’ with a median lethal concentration of more than 1000 mg/L after 96 h. The biodegradation rate was recorded as higher than 60% for both surfactants within 28 days, demonstrating their readily biodegradable nature. Considering these attributes, biocompatible and biodegradable surfactants derived from orange peel emerge as a promising and sustainable alternative for oil spill remediation.

## 1. Introduction

Oil is one of the primary sources of energy, especially in the modern world. The exploration of oil wells has been associated with the risk of oil spill incidents, with Deepwater Horizon in 2010 and Exxon Valdez in 1989 as notable examples [1,2]. Oil spills pose diverse concerns as they can directly affect health, safety, the environment, and the economy [3,4]. Oil spill remediation significantly minimises the impacts of spilt oil on the environment [5,6]. Various mitigation measures have been investigated and practised since these significant oil spill incidences occurred, such as the Deepwater Horizon in the Gulf of Mexico and the Exxon Valdez in Alaska [7,8]. Therefore, effective oil spill remediation is required to combat environmental issues effectively. The methods for oil spill remediation include physical, chemical, biological, and thermal approaches [9,10]. Chemical methods using dispersants for oil spill remediation are widely recognized. Chemical methods use surfactants that can be dissolved in any organic solvent, and they reduce the surface tension of the oil-water phase and break down the oil slicks into smaller oil droplets, thereby increasing the dispersion of crude oil [11]. Corexit was the most effective oil spill dispersant among all chemical dispersants. However, Corexit contained toxic surfactants and 2-butoxyethanol, a carcinogenic compound [12,13]. When Corexit was used in the Deep Water Horizon oil spill, it was observed that significant harm was caused to the marine biota due to interactions with crude oil, which enhanced the toxicity and depleted the oxygen level [14]. Commercial anionic surfactants such as linear alkylbenzene sulfonates (LAS) can cause skin irritation and respiratory issues. They also inhibit the resistance of marine life in combating various environmental challenges and reproduction processes. In contrast, cationic surfactants are determined to be more toxic [15,16]. Consequently, the emphasis has turned to a new class of dispersants using renewable raw materials with higher emulsifying ability and lower toxicity [17].

Oranges are among the most popular fruits in the world, and there is a high demand for oranges resulting in the production of vast quantities of orange peel (OP) [18]. The waste from OP in many industrial processes is causing several environmental issues [18]. Numerous research studies have been conducted to determine the chemical structure of OP [19] and convert the identified elements into value-added products to prevent environmental problems [20]. OP consists of free sugars (30–36%), cellulose (18–20%), hemicellulose (14–16%), pectin (20–22%), lignin (5–7%), protein (up to 8%), and essential oils (0.3–0.5%) [21,22]. Researchers are increasingly interested in the uses of cellulose and its plant-based derivatives [23]. The cellulose extraction from lignocellulosic compounds involves physical, chemical, and biological techniques, or a combination of these, to break down the cellulose-hemicellulose-lignin compound [22]. The chemical method was selected, and conventional organic solvents were chosen as the most popular solvents for extracting natural organic compounds such as carotenoids. However, their high volatility and toxicity must be addressed [20].

The emergence of the “Green Chemistry” concept has changed how academics and industry develop chemical procedures, highlighting an increase in environmental awareness and interest in sustainable development over the last several decades [24]. In this regard, researchers have focused on developing environmentally friendly extraction techniques. ILs are gaining higher interest due to their outstanding properties and are a fantastic substitute for conventional organic solvents [25,26]. ILs can even be recycled to be utilized multiple times, making it a feasible and efficient system compared to other organic solvents. From Cellulose Degree of polymerization (DP) and other analyses, alkali-based pre-treatments are recommended as they can distinctively extract pectin [27]. Wang et al., 2018 mentioned using acid chlorides with tertiary bases such as pyridine is a common method used to produce cellulose fatty acid esters with various aliphatic chain lengths [28]. The inefficiency of organic solvents and the generation of a significant amount of toxic waste have detrimental effects on health, safety, and the environment. As a result, there has been a strong emphasis on finding ways to reduce solvent consumption and adopt more environmentally friendly solvents [29]. To effectively remediate oil spills, it is necessary to utilize ILs to synthesise cellulose from OP while assuring reduced toxicity [30].

In this study, OP was used to synthesize the surfactant by using organic solvent (DMAc/LiCl) and IL (1-Butyl-3-methylimidazolium chloride (BMIMCl), respectively. The comparative performance of the synthesized surfactant was evaluated through surface tension measurement, emulsion stability, dispersant effectiveness, and the size of dispersed oil droplets. For the secure use of synthesized surfactant in the environment, toxicity and biodegradability studies were also performed.

## 2. Results

### 2.1. Surface Tension

**Figure 1:**
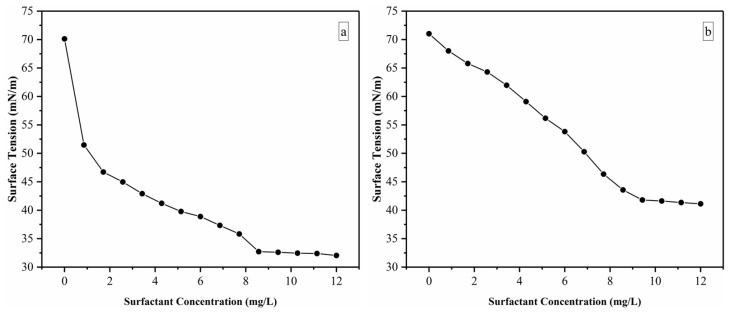
Surface tension against surfactant concentrations, (**a**) SOPIL (**b**) SOPOS.

The surface tension method determined the critical micelle concentration (CMC) values of synthesized surfactants. The CMC values of SOPIL and SOPOS surfactant were 8.57 mg/L and 9.42 mg/L, respectively, as shown in Figure 1a,b. It was evident that as the concentration of the surfactant mixture increased, the surface tension decreased until it reached a plateau region with distinct breakpoints. Pillai et al. 2022) described that the surface tension of the solution reduced as the concentration of surface-active ionic liquids (SAILs) increased. They added by stating that this was because the SAIL molecules adsorbed on the air-water interface and lowered the system’s free energy. The surface tension decreased until it became almost constant because no more SAILs molecules could adsorb on the surface and became saturated [31]. Since Dib et al. 2021) mentioned that SAILs can self-segregate in solution and behave similarly to surfactants, it can be deduced that the SOPIL surfactant could reduce surface tension [32]. Beyond the CMC point, the concentration of SOPIL surfactants did not affect the surface tension values. This was due to the saturated monolayer coverage of the component being formed at the air-water interface [33]. This trend agreed with the previous studies [12,34]. The study conducted by Wang et al., 2022 showed the effect of different sodium dodecyl benzene sulfonate (SDBS) concentrations towards the surface tension of oil-based foam systems. The results demonstrated that the surface tension of the foam system decreases as concentration increases [35]. However, a more regular plateau region was observed with the increase of surfactant concentration, similar to the trend of our surface tension results, as shown in Figure 1a,b. However, SOPIL surfactant exhibited a sharper decrease in surface tension, as shown in Figure 1a. Besides that, the lower CMC values illustrated that SOPIL surfactant has better capability in decreasing the surface tension than SOPOS surfactant. This is because a lower CMC indicates that less surfactant is required to achieve a certain degree of emulsification or solubilization which can lead to cost savings and reduce environmental impact. A lower CMC value also indicates an improvement in the effectiveness as it indicates that the surfactant can penetrate and remove oil much more easily.

### 2.2. Emulsion Stability

**Figure 2:**
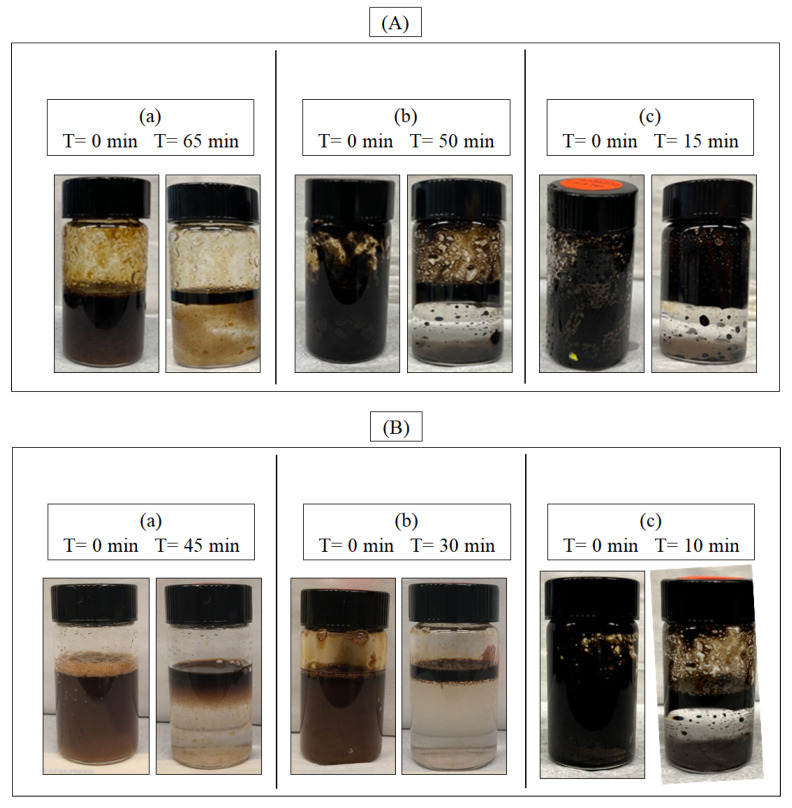
Emulsification of (**A**) SOPIL with (**a**) Bonny (light); (**b**) Arab (medium); (**c**) Doba (heavy) crude oils and (**B**) SOPOS with (**a**) Bonny (light); (**b**) Arab (medium); (**c**) Doba (heavy) crude oils. The surfactant concentration was 25 wt%.

Emulsion stability refers to the ability of a surfactant to resist breaking and remain homogeneous for a certain period. Bonny (light), Arab (medium), and Doba (heavy) crude oils with simulated seawater (1:10 by volume) were emulsified by pure SOPIL and SOPOS dispersants to evaluate the stability of the emulsions as shown in Figure 2. The surfactant concentration used for all samples was 25 wt%. The crude oil and dispersant were kept at a constant 1:1 volume ratio. The results are shown in Figure 2A,B for SOPIL and SOPOS, respectively. All samples were imaged immediately after emulsion preparation by vortex mixer at 0 min and again at 5 min intervals. Initially, all samples comprised a stable black or brown homogeneous emulsion. However, after 65 min, 50 min and 15 min, the emulsions destabilized respectively (from light to heavy crude oils) for SOPIL as demonstrated in Figure 2(Aa–Ac).

Nevertheless, the emulsion destabilized for SOPOS after 10 min for Doba, 30 min for Arab and 45 min for Bonny. They formed two transparent, distinct layers (unstable), whereby the upper phase was oil-rich (dark brown or black), and the bottom phase was water-rich and lighter in colour. Surfactants are amphiphilic compounds and have both hydrophilic and hydrophobic ends. One of the roles of a surfactant is to lower the interfacial tension between two immiscible liquids [36,37]. It is reported that surfactants enhanced emulsion stability by acting as a barrier against the coalescence of oil droplets [12]. Also, emulsion stability time varies depending on the type of crude oil. For example, light crude oils have lower viscosity and are more easily dispersed and form more stable emulsions than middle and heavy crude oils.

### 2.3. Dispersant Effectiveness

**Figure 3:**
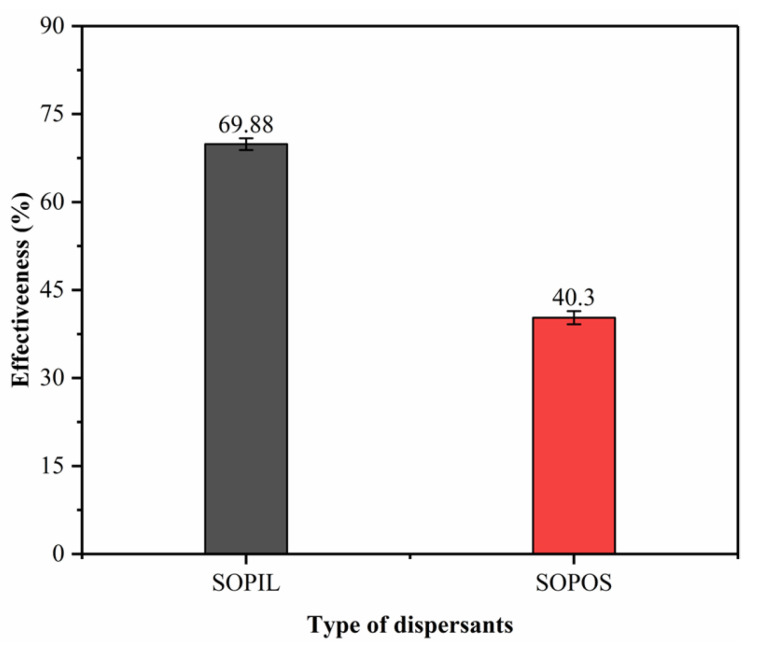
Dispersant Effectiveness of SOPIL and SOPOS surfactants.

The dispersant effectiveness of both SOPIL and SOPOS surfactants is shown in Figure 3. The oil used in this experiment was Arab (medium) crude oil, and the concentration of surfactant used was 25 wt% with a DOR of 1:25 by volume. The dispersant performed better between 1:1 and 1:25 DOR. To corroborate these findings, Baharuddin et al. 2020) stated that at higher DOR, higher values of effectiveness were achieved [38]. Based on the findings, SOPIL surfactant was more effective than SOPOS surfactant, reaching 69.88% compared to SOPOS surfactant with only 40.30%. This finding is consistent with the literature, where a dispersant composed of a Tween-80 blend and an ionic liquid had an effectiveness of 63.83% for Ratawi (medium) crude oil [12]. Baharuddin et al. 2020) reported an IL-based dispersant effectiveness of 70.75% for Ratawi (medium) crude oil [38]. Hence, with these similar findings, it can be deduced that the SOPIL surfactant dispersant effectiveness was good and acceptable compared to the SOPOS surfactant. Many reasons can contribute to these findings. It may be because surfactants prepared from ionic liquids (ILs) have higher purity than organic solvents. Given that ILs are free from impurities typically present in organic solvents. Besides that, one possible reason is surfactants prepared from ILs have been shown to have a higher surface activity than those prepared from organic solvents, which leads to better wetting and spreading properties which are critical for more effective oil dispersion.

### 2.4. Optical Microscopy

**Figure 4:**
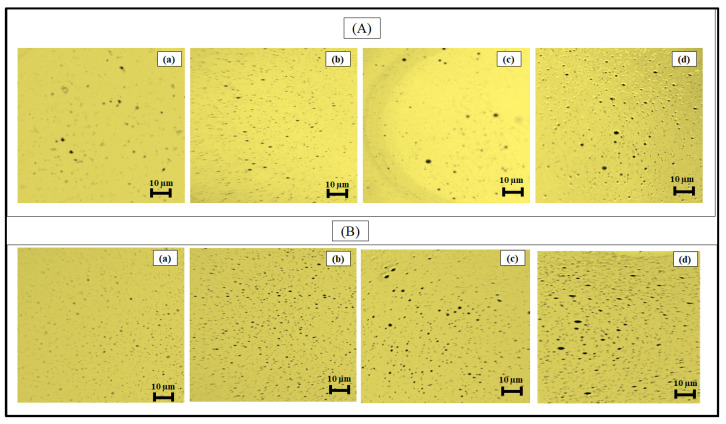
Optical micrographs of dispersed Arab crude oil using DOR ratios of :(**a**) 1:1; (**b**) 1:25; (**c**) 1:50; and (**d**) 1:100 for (**A**) SOPIL and :(**a**) 1:1; (**b**) 1:25; (**c**) 1:50; and (**d**) 1:100 for (**B**) SOPOS.

Optical microscopy images of the dispersed Arab crude oil droplets were recorded to examine the dispersion of crude oil. Figure 4A,B illustrated the optical micrographs taken with 25 wt% SOPIL and SOPOS surfactants at various DORs (*v*/*v*) of 1:1, 1:25, 1:50 and 1:100. From Figure 4. It was noted that the average diameter of dispersed oil droplets decreases when the DOR increases from 1:100 to 1:1. Quantitatively, the average diameter of dispersed oil droplets was tabulated in Table 1 below. Reducing oil droplet sizes increased emulsion stability performance, and ultimately the dispersant effectiveness was increased [39]. These results were consistent with research from Afsher et al. 2022), who found that size of oil droplets increases as DOR decrease from 1:1 to 1:100 [40]. Two main reasons can contribute to support these findings. Firstly, the size reduction could be due to the surfactant mixture being adsorbed rapidly at the oil-water interface and decreased interfacial tension (IFT), causing the crude oil to be broken down into tiny droplets. Besides that, as DOR increased, a sufficient quantity of surfactant was available to disperse the oil into smaller droplets.

### 2.5. Toxicity Evaluation

The acute toxicity of synthesized SOPIL and SOPOS surfactant against zebrafish (D. rerio) was evaluated by performing fish toxicity experiments. At first, the limit test was conducted at a concentration of 100 mg/L. After four days, there was no dead fish in both fish tank, indicating that the median lethal concentration (LC_50_) was higher than 100 mg/L. According to US Environmental Protection Agency, the aquatic toxicity scale for laboratory-generated aquatic toxicity data classified LC_50_ (the concentration at which half population died) greater than 100 mg/L as practically non-toxic. After that, the experiments were repeated at different concentrations ranging up to 1000 mg/L with ten separate tanks containing ten fish. The fish were observed in the tanks, and if any were found dead, they were promptly and appropriately removed. Our findings determined that no fish died even when the concentration was 1000 mg/L, as mentioned in Table 2. Therefore, the SOPIL surfactant was classified as ‘Relatively harmless’ according to Passino and Smith toxicity scale [41].

On the other hand, the acute toxicity of synthesized SOPOS surfactant against zebrafish was also evaluated by performing the same experimental procedure. As the concentration increased up to 400 mg/L, it was observed that one fish died. At 450 mg/L, the mortality percentage was 50%, indicating that 450 mg/L was the median lethal concentration of SOPOS surfactant. Therefore, the SOPOS surfactant was classified as ‘Practically harmless’ according to Passino and Smith scale. In addition, the mortality rate was 100%, in which all fish died when the concentration increased to 500 mg/L, as demonstrated in Table 2. From the findings, it was deduced that SOPIL surfactant has a lower level of acute toxicity when compared to SOPOS. This is because of the toxic nature of the used organic solvent in SOPOS. When DMAc is combined with LiCl, it becomes highly toxic, corrosive, and volatile, increasing health and safety risks and concerns [42,43].

### 2.6. Biodegradability

Biodegradation is when microorganisms convert organic compounds to less complex chemical structures [44]. Material biodegradation is still an issue as it can affect the ecosystem [45]. Therefore, this investigation is vital to determine whether persistent compounds can be removed from the environment [6]. Organization for Economic Co-operation and Development (OECD) defines a substance as a biodegradable material if 60% of the original amounts degrade aerobically in 28 days [46]. In this experiment, a closed bottle test (CBT, OECD 301D, 1992) was performed for the biodegradation rate of both SOPIL and SOPOS surfactants. Based on our findings, both SOPIL and SOPOS surfactants achieved a biodegradation percentage of 80.64% and 65.97%, respectively, higher than 60%, as presented in Table 3. From the Table, it can be found that SOPIL surfactant showed a higher biodegradation percentage than SOPOS surfactant. The findings were compared to previously reported commercial surfactants in the present study. Brakstad et al., conducted a study on the biodegradability of DOSS in seawater. According to their results, the degradation rate of DOSS was observed to be 16% over 54 days [47]. The biodegradability of conventional dispersants such as Corexit 9500A ranges from 10% to 20% after 30 days [48]. Besides that, Prince et al. 2015) studied three conventionally used commercial dispersants: Corexit 9500A, Dasic Slickgone and Finasol OSR52. The biodegradation rate for the three of them was 32% after 28 days [49].

## 3. Materials and Methods

### 3.1. Materials

IL, 1-Butyl-3-methylimidazolium chloride (BMIMCl), sodium hydroxide (NaOH) pellets, acetic acid (CH_3_COOH) solution, hydrochloric acid (HCl) solution, Dimethylacetamide (DMAc), Lithium Chloride (LiCl), Octanoyl chloride (C_8_H_15_ClO), anhydrous pyridine, ethanol (C_2_H_5_OH) were obtained from the Sigma-Aldrich. 24.95 g/L sodium chloride (NaCl) solution, 5.23 g/L magnesium chloride (MgCl_2_) solution, 3.835 g/L sodium sulphate (Na_2_SO_4_) solution were used to prepare simulated seawater [50]. The Bonny (light), Arab (middle) and Doba (heavy) crude oil were primarily used for emulsion stability and dispersant effectiveness tests. All these crude oils were obtained from Malaysia Mutual Aid Group’s Petroleum industry. The water sample used for the biodegradability test was obtained at the gas district cooling plant, Universiti Teknologi PETRONAS, Perak, Malaysia. Zebrafish (*Danio rerio*) were bought and obtained from local fishermen in Perak, Malaysia, for toxicity.

### 3.2. Synthesis of Surfactants

#### 3.2.1. Preparation of Orange Peel Powder

Fresh orange was bought from the local supermarket in Melaka, Malaysia. The orange peels were washed to remove any impurities. The washed orange peels were then cut into smaller pieces before putting into the oven for 24 h at 80 °C to remove the moisture. After 24 h, the dried orange peel pieces were ground by a grinder and filtered through a 50 µm sieve shaker. The powdered orange powder was later dried in the oven for 2 h at 100 °C. The process of preparation of orange peel powder is described in Figure 4.

#### 3.2.2. Pre-Treatment and Delignification

**Figure 5:**
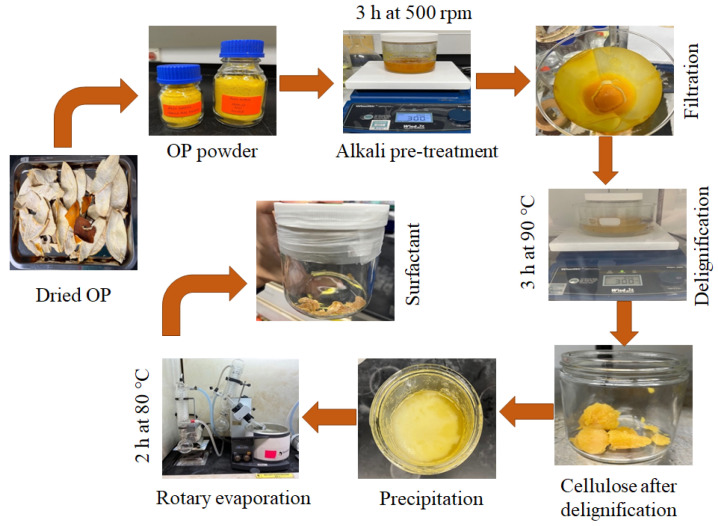
Synthesis of surfactant from Orange Peel.

Figure 5 demonstrates the pre-treatment and delignification procedure for the orange peels. The powder (4 g) was pre-treated with 2 wt% sodium hydroxide (NaOH) solution for 3 h at 500 rpm to remove impurities and pectin, as shown in Figure 5. Alkali pre-treatment was recommended as they extract pectin distinctively [27]. The pH of the peel powder was determined to ensure that the powdered pH value was in the neutral range. Once the pH values were satisfied, filtration was done to remove the NaOH solution, as shown in Figure 5. After that, the delignification process was performed using 70 wt% acetic acid (CH_3_COOH) and 0.027 M HCl. Both CH_3_COOH and HCl prepared were poured into the vial containing filtered solid. The vial was placed on a magnetic stirrer in a silicon bath at 90 °C for 3 h (Figure 5). After delignification, filtration was performed to remove lignin and the remaining impurities. The pH of the peel powder was determined again after this step. Furthermore, the pre-treated and delignified material was ensured to be within the neutral pH value range.

#### 3.2.3. Cellulose Esterification

The cellulosic material in Figure 5 was mixed with two different types of solvents: organic solvent (OS) and IL-based solvent. Cellulosic material dissolved in IL (1-butyl-3-methylimidazolium chloride [BMIM] or OS (DMAc/LiCl) at 80 °C for 8 h at 700 rpm. It has been reported in various literature that the ionic liquid 1-butyl-3-methylimidazolium (BMIMCl) or BMIMCl/DMF mixture can easily dissolve the lignocellulosic biomass, including wood biomass. We have selected this as a reaction medium because the BMIMCl/DMF mixture allows the complete dissolution of cellulose, followed by a fast reaction with acetic anhydride [51]. After 8 h, cellulose esterification reaction was conducted by reaction between the solvents dissolved cellulosic material and 10 mmol octanoyl chloride catalysed by 10 mmol anhydrous pyridine. The reaction mixture was heated for another 8 h at 90 °C at 700 rpm. In the meantime, cold distilled water was prepared when the reaction was almost completed, e.g., after 14 h. After the completion of the reaction, cold distilled water was poured into the reaction solution (Figure 5). The cold water precipitated the product known as “surfactant”, and filtration was done to obtain a surfactant free of impurities and other solutions. After that, the washing of surfactant with ethanol was carefully conducted about 2 to 3 times, followed by filtration to remove the remaining ethanol. The pure surfactants were obtained after evaporation by a rotary evaporator at 80 °C for 2 h, as shown in Figure 5. As shown in Figure 5, the final product was confirmed by nuclear magnetic resonance spectroscopy (Bruker Avance III NMR (Bruker, Billerica, MA, USA) at 500 MHz) NMR (Supplementary Materials, Figures S2–S5) and Fourier Transform Infrared Spectroscopy (Shimadzu IR-Tracer-100, Shimadzu, Kyoto, Japan) FTIR (Supplementary Materials, Figure S1). The confirmation of cellulose acylation was achieved after the esterification reaction by integrating the distinctive signals of fatty acid protons (1.043 to 2.89 ppm) and the cellulose backbone (3.165–4.124 ppm, carbohydrate protons), and these results are corroborated by the previously published data [52]. The acronym SOPIL and SOPOS were used for surfactants prepared with IL and OS, respectively.

### 3.3. Characterization of Surfactants

The characterization of the synthesized surfactants was conducted at room temperature and pressure of 25 °C and 1 atm, respectively.

#### 3.3.1. Measurement of Surface Tension

At room temperature, the surface tension of the surfactants was determined using a tensiometer (DCAT 15, Data Physics, Stuttgart, Germany). A stock solution of synthesized surfactants with a concentration of 25 g/L was prepared by dissolving 0.5 g of surfactant in 20 mL of distilled water. Three measurements were taken during each experiment, with an uncertainty of ±0.02 mN/m associated with each measurement. To calibrate the instrument, the surface tension of water was measured, and its value was 71.99 mN/m.

#### 3.3.2. Emulsion Stability

The simulated seawater-crude oil blend was prepared on a ratio of 1:10 by mixing 1 mL of each crude oil with 10 mL of simulated seawater. Then, 1 mL of 25% *w*/*w* dispersant was added to the blended mixture. A vortex meter was used to stir the sample at 3000 rpm for 3 min to form the emulsion [12]. After that, the samples were left undisturbed, and observation was done at various intervals until an unstable emulsion (2 distinct layers) was observed.

#### 3.3.3. Dispersant Effectiveness

To evaluate dispersant effectiveness of the dispersant, a baffled flask test (BFT) was conducted. 120 mL of simulated seawater was poured into the baffled flask, followed by 100 µL crude oil. After this, 4 µL of dispersant was added to achieve the dispersant-to-oil ratio (DOR) 1:25 [12]. The amount of dispersants added was manipulated at a constant volume of crude oil and temperature at 25 °C. After that, the flask containing the dispersant-oil-simulated seawater mixture was placed in an incubator shaker at a rate of 200 rpm for 10 min, and left to stand for 10 min. The next step was to collect and extract 30 mL of dispersed sample from the flask using DCM. The UV-Vis Spectrophotometer was used to determine the absorbance values at wavelengths (λ) of 340 nm, 370 nm, and 400 nm.

The area was calculated by trapezoidal rule from [12] using Equation (1).

(1) $$\mathrm{Area}=\frac{\left({\mathrm{Abs}}_{340}+\;{\mathrm{Abs}}_{370}\right)\times 30}{2}+\frac{\left({\mathrm{Abs}}_{370}+\;{\mathrm{Abs}}_{400}\right)\times 30}{2}$$

where Abs_340_, Abs_370_ and Abs_400_ are the absorbance measurements at the wavelength (λ) of 340 nm, 370 nm and 400 nm, respectively. Once the area values were obtained, the total oil dispersed (in grams) in the water column was determined from [12] with Equation (2) below.

(2) $$\mathrm{Total}\;\mathrm{oil}\;\mathrm{dispersed}\;\left(g\right)=\frac{\mathrm{Area}\;}{\mathrm{Calibration}\;\mathrm{curve}\;\mathrm{gradient}}\times {\;V}_{\mathrm{DCM}}\times \frac{V_{\mathrm{tw}}}{V_{\mathrm{ew}}}$$

where

V_DCM_ = Volume of dichloromethane (DCM) extract (L)

V_tw_ = Total volume of synthetic seawater in the baffled flask (L)

V_ew_ = Volume of synthetic water extracted for dispersed oil content (L)

Lastly, Equation (3) calculated and determined the dispersion effectiveness [12].

(3) $$\mathrm{Dispersion}\;\mathrm{effectiveness}\;\left(\%\right)=\frac{\mathrm{Total}\;\mathrm{oil}\;\mathrm{dispersed}}{\rho_{\mathrm{oil}}\times {\;V}_{\mathrm{oil}}}\times 100$$

where

$\rho_{\mathrm{oil}}$ = Density of test oil in g/L

$V_{\mathrm{oil}}$ = Volume of crude oil added to baffled flask (L)

#### 3.3.4. Optical Microscopy

An optical microscope (Zeiss optical microscope, Zeiss, Jena, Germany) was used to capture and determine the size of dispersed oil droplets at different time intervals. A drop of the mixture was taken by a syringe and placed on a glass microscope slide. The experiment was repeated at different DORs ratios, which are 1:1, 1:25, 1:50 and 1:100 *v*/*v*. The magnification was set at 50 µm to obtain clear pictures of dispersed oil droplets.

#### 3.3.5. Acute Fish Toxicity Analysis

The toxicity experiment was conducted using zebrafish to determine the median lethal concentration (LC_50_). Zebrafish were put inside a container with their survival conditions set as constant such as 90% dissolved oxygen level, water pH of 8.0 and at room temperature (23 °C to 26 °C) [53]. Ten fish were taken for each of the dispersant concentrations introduced to the fish containers for toxicity analysis. The variable in this acute toxicity experiment was the concentration of dispersants. The concentrations were, 200 mg/L, 300 mg/L, 400 mg/L, 500 mg/L, 700 mg/L, 850 mg/L, 900 mg/L, 950 mg/L, and 1000 mg/L. The number of dead and alive fish was observed at an interval of 24 h for four days. The toxicity classification was based on the scale developed by Passino & Smith, 1987 as mentioned in Supplementary Materials Table S1 [41].

#### 3.3.6. Biodegradability

The biodegradability experiments were conducted to determine the time taken for the substance to degrade under aerobic conditions [6]. The Closed Bottle Test (CBT, 301D, 1992) was conducted to assess biodegradability, following the guidelines of the Organization for Economic Cooperation and Development (OECD) 301 [46]. A seedling water sample was obtained from the gas district cooling plant at the Universiti Teknologi PETRONAS, Perak, Malaysia. At the same time, stock solutions for mineral medium were prepared accordingly (Supplementary Materials Table S2). The mineral medium was prepared by mixing 1 mL of stock solutions A, B, C and D (Supplementary Materials Table S2) with 800 mL water and was then made up to 1 L in a volumetric flask. BOD bottles (500 mL) were filled with mineral medium, inoculum (containing 10^6^ cells/mL bacteria), and test substance having 10 mg/L concentration. All these samples were placed in an OxiTop system (WTW GmbH, Weilheim, Germany). The experiment was conducted for 28 days at 20 °C. A blank sample without a test substance was also conducted. In determining the percentage of biodegradation, Equation (4) from [6] was used.

(4) $$\%\mathrm{Degradation}=\frac{\mathrm{BOD}\;\left(\frac{{\mathrm{mg}\;O}_{2}}{\mathrm{mg}\;\mathrm{test}\;\mathrm{substance}}\right)}{\mathrm{ThOD}\;\left(\frac{{\mathrm{mg}\;O}_{2}}{\mathrm{mg}\;\mathrm{test}\;\mathrm{substance}}\right)}\times 100$$

## 4. Conclusions

The biobased surfactants from orange peels were synthesized and utilized for oil spill remediation. The CMC values of SOPIL and SOPOS surfactant were 8.57 mg/L and 9.42 mg/L, respectively. Emulsification of synthesized surfactants was conducted using light, medium and heavy crude oils, and the results revealed that SOPIL was more stable than SOPOS. The dispersant effectiveness of the SOPIL and SOPOS surfactants was determined to be 69.88% and 40.30%, respectively. The size of dispersed oil droplets also decreased as a dispersant-to-oil ratio (DOR) increased from 1:1 to 1:100. The acute toxicity experiment against zebrafish determined that SOPIL had LC_50_ more than 1000 mg/L, which indicated that it was relatively harmless. The biodegradation values of SOPIL and SOPOS surfactants were 80.64% and 65.97%, respectively. It can be concluded that the surfactant synthesized from orange peel using IL as an environmentally benign reaction medium provided higher dispersion effectiveness for oil spill remediation than surfactant synthesized using an organic solvent”.

## Figures and Tables

**Table 1:** Optical microscopy analysis of SOPIL and SOPOS surfactants.

DOR	Diameter (mm)			Average Diameter (mm)
	1	2	3	
SOPIL surfactant				
1:100	0.653	0.254	0.558	0.489
1:50	0.175	0.230	0.601	0.335
1:25	0.230	0.143	0.421	0.265
1:1	0.090	0.125	0.207	0.141
SOPOS surfactant				
1:100	0.583	0.761	0.672	0.672
1:50	0.603	0.712	0.342	0.519
1:25	0.385	0.496	0.583	0.488
1:1	0.238	0.333	0.341	0.304

**Table 2:** Acute fish toxicity of the newly synthesized surfactants.

Concentration (mg/L)	Number of Tested Fish	Number of Dead Fish (after 4 Days)	Mortality Percentage (%)
SOPIL surfactant			
100	10	0	0
200	10	0	0
300	10	0	0
400	10	0	0
500	10	0	0
600	10	0	0
700	10	0	0
800	10	0	0
900	10	0	0
950	10	0	0
1000	10	0	0
SOPOS surfactant			
100	10	0	0
200	10	0	0
300	20	0	0
375	10	1	10
400	10	3	30
425	10	4	40
450	10	5	50
480	10	8	80
500	10	10	100

**Table 3:** Biodegradability analysis of SOPIL and SOPOS surfactants.

Test Materials	R1	R2	R3	R4	%Biodegradation ± SD ^a^
SOPIL	77.27	79.32	82.72	83.25	80.64 ± 2.84
SOPOS	63.88	65.73	67.55	66.72	65.97 ± 1.58
Reference ^b^	94.12	94.97	95.78	92.87	94.44 ± 1.24

^a.^ The average biodegradation of four replicas (R). ^b^ Sodium acetate was used as a reference material.

## Data Availability

As this study is currently part of an ongoing project, we are unable to share the raw and processed data required for replicating these findings at this time.

## Supplementary Materials

The following supporting information can be downloaded at: https://www.mdpi.com/article/10.3390/molecules28155794/s1, Figure S1: FTIR Spectra of SOPIL and SOPOS; Figure S2: NMR Spectra of SOPIL; Figure S3: NMR Spectra of SOPOS; Figure S4: Expected SOPIL structure; Figure S5: Expected SOPOS structure; Table S1: Toxicity scale developed by Passino & Smith, 1987; Table S2: Preparation of Stock Solutions for Mineral Medium.
