# Supplementary material for: Biocompatible and Biodegradable Surfactants from Orange Peel for Oil Spill Remediation
Source: Molecules. 2023 Aug 1;28(15):5794. doi: 10.3390/molecules28155794 (PMC10421384; doi:10.3390/molecules28155794)
Supplement: Supplementary file 1 [file molecules-28-05794-s001.zip › molecules-2508979-supplementary.pdf]

Supplementary Data

Biocompatible and Biodegradable Surfactants from Orange Peel for Oil Spill Remediation

Wang Peng Soon[1], Aqeel Ahmad[1], Masooma Nazar[1], Anisa Ur Rahmah[2], Muhammad Moniruzzamana [1,3]*

[1] Department of Chemical Engineering, Universiti Teknologi PETRONAS, 32610, Bandar Seri Iskandar, Perak, Malaysia; peng_18002311@utp.edu.my; masooma.syed14@gmail.com; aqeelahmadms@gmail.com

[2] Department of Chemical Engineering, Universitas Muhammadiyah Surakarta, Pabelan, Kartasura, Sukoharjo, 57162, Indonesia; aur744@ums.ac.id

[3] Center of Research in Ionic Liquids (CORIL), Universiti Teknologi PETRONAS, 32610, Bandar Seri Iskandar, Perak, Malaysia

* Correspondence: m.moniruzzaman@utp.edu.my

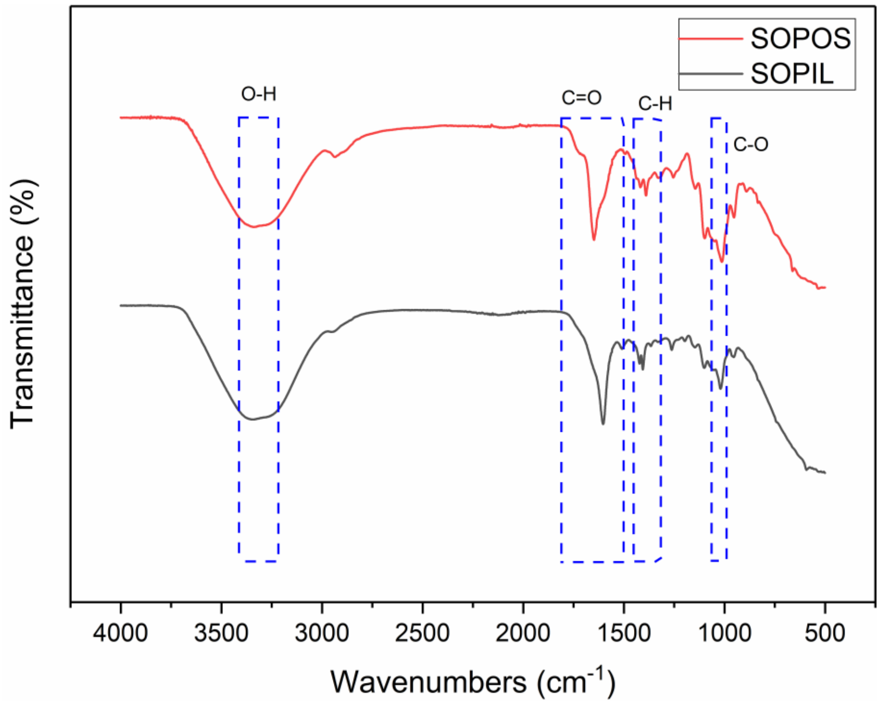

Figure S1: FTIR Spectra of SOPIL and SOPOS

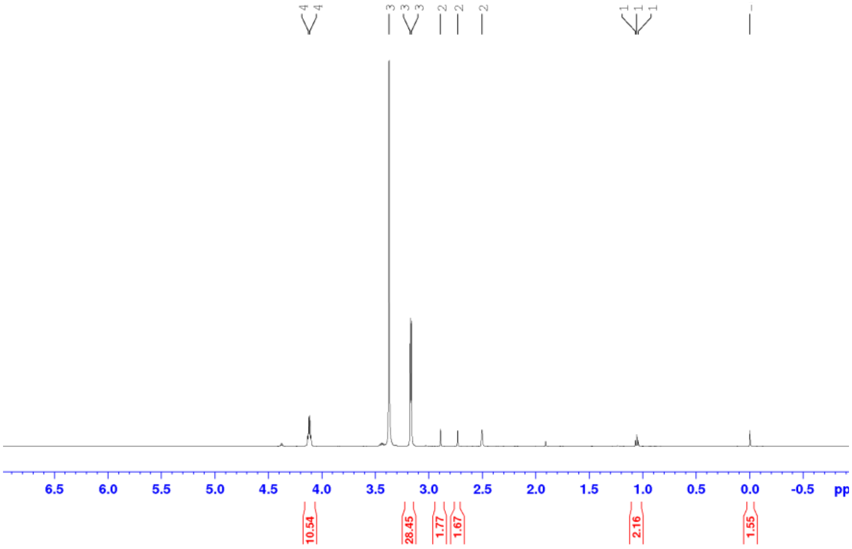

Figure S2: NMR Spectra of SOPIL

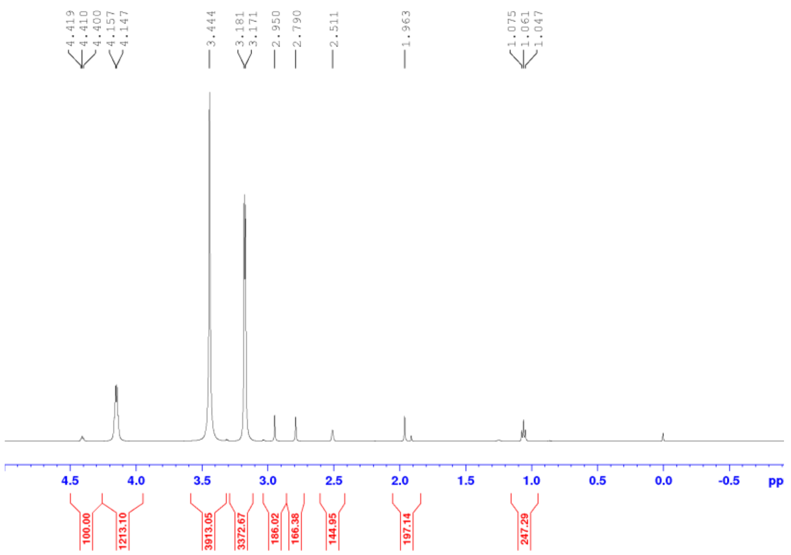

Figure S3: NMR Spectra of SOPOS

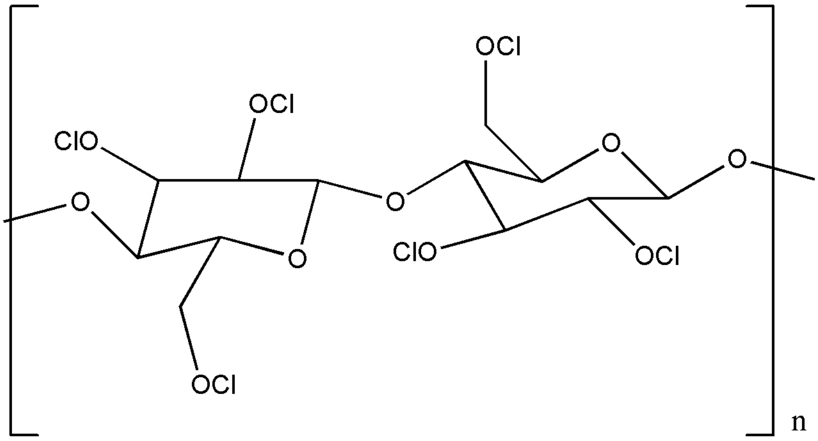

Figure S4: Expected SOPIL structure

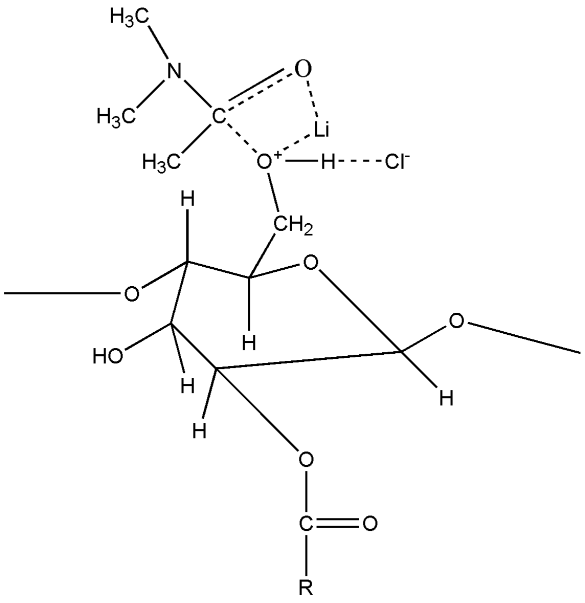

Figure S5: Expected SOPOS structure

Table S1: Toxicity scale developed by Passino & Smith, 1987

**Concentration (mg/L)**	**Toxicity Scale**
Less than 0.01	Super toxic
0.01 to 0.1	Extremely toxic
0.1 to 1	Highly toxic
1 to 10	Moderately toxic
10 to 100	Slightly toxic
100 to 1000	Practically harmless
Greater than 1,000	Relatively harmless

Table S2: Preparation of Stock Solutions for Mineral Medium

**Stock solution**	**Required reagent(s)**	**Mass required (g)**	**Descriptions**
A	Potassium dihydrogen orthophosphate, KH_2PO_4	2.13	Dissolve in water and make up to 0.25 L. The pH of the solution should be 7.4
	Dipotassium hydrogen orthophosphate, K_2HPO_4	5.44	
	Disodium hydrogen orthophosphate dihydrate, $Na_2HPO_4.2H_2O$	8.35	
	Ammonium chloride, NH_4Cl	0.13	
B	Calcium chloride dihydrate, $CaCl_2.2H_2O$	9.10	Dissolve in water and make up to 0.25 L.
C	Magnesium sulphate heptahydrate, $MgSO_4.7H_2O$	5.63	Dissolve in water and make up to 0.25 L.
D	Iron (III) chloride hexahydrate, $FeCl_3.6H_2O$	0.06	Dissolve in water and make up to 0.25 L.
